# Higher-dimensional performance of port-based teleportation

**DOI:** 10.1038/srep33004

**Published:** 2016-09-08

**Authors:** Zhi-Wei Wang, Samuel L. Braunstein

**Affiliations:** 1 Tang Aoqing Honors Program in Science, College of Physics, Jilin University, Changchun, 130012, People’s Republic of China; 2 Computer Science and York Centre for Quantum Technologies, University of York, York YO10 5GH, United Kingdom

## Abstract

Port-based teleportation (PBT) is a variation of regular quantum teleportation that operates without a final unitary correction. However, its behavior for higher-dimensional systems has been hard to calculate explicitly beyond dimension *d* = 2. Indeed, relying on conventional Hilbert-space representations entails an exponential overhead with increasing dimension. Some general upper and lower bounds for various success measures, such as (entanglement) fidelity, are known, but some become trivial in higher dimensions. Here we construct a graph-theoretic algebra (a subset of Temperley-Lieb algebra) which allows us to explicitly compute the higher-dimensional performance of PBT for so-called “pretty-good measurements” with negligible representational overhead. This graphical algebra allows us to explicitly compute the success probability to distinguish the different outcomes and fidelity for arbitrary dimension *d* and low number of ports *N*, obtaining in addition a simple upper bound. The results for low *N* and arbitrary *d* show that the entanglement fidelity asymptotically approaches *N*/*d*^2^ for large *d*, confirming the performance of one lower bound from the literature.

Port-based teleportation (PBT)^1^ is a variation of conventional quantum teleportation, which can be used as a universal processor. In PBT, Alice (the sender) and Bob (the receiver) share *N* pairs of entangled quantum states (without loss of generality we assume these to be maximally entangled). Then Alice performs a joint measurement (a positive operator valued measurement, POVM, 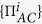) on her resource and the input quantum state 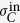 that she wishes to teleport. Finally, Alice tells Bob the measurement outcome *i* ∈ {0, 1, …, *N*}. If *i* = 0 the teleportation is considered to have failed, otherwise, Alice’s input state 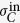 will have been teleported to the *i*th entangled partner (or port) in Bob’s possession, see Fig. 1. Unlike conventional teleportation^2^, PBT does not require any corrective unitary operation at Bob’s side other than to discard the unused ports, however, it achieves this at the cost of requiring more entanglement, while simultaneously realising only a finite probability of success.

The lack of a final correcting unitary for the PBT protocol has led to a number of important theoretical implications, including universal quantum processing, non-local processing, etc. (for a more complete discussion of applications see refs 3 and 4). Despite its clear theoretical importance explicit calculations of the achievable performance of PBT are largely restricted to low dimensional systems^1^ (*d* = 2 with arbitrary *N*, and *d* = 3 for *N* ≤ 6). There are several papers giving bounds to the performance of PBT more generally^3^,^5^,^6^,^7^, but they do not explicitly calculate the achievable fidelity (or other performance measures) for higher dimensional systems. In addition, some bounds become trivial in higher dimensions, for example some lower bounds become negative for higher dimensional systems^3^,^5^,^6^,^7^. Thus it is not clear that we may rely on these bounds for evaluating the theoretical performance of PBT in its manifold applications.

Since a *d*-dimensional quantum system can be written in terms of 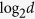 qubits, we might naively expect that the PBT of a *d*-dimensional system would be equivalent to 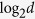 repetitions of the PBT protocol on this many qubits. This reasoning is incorrect for at least two reasons. First, PBT does not operate with either unit success probability or with unit fidelity, so this decomposition would not yield the same performance except possibly in the asymptotic limit of an infinite number of ports. Second, in the most general scenario, the *N* ports at Bob’s side could be non-locally distributed among *N* Bob’s (Bob_1_, Bob_2_, etc.). In this scenario, a single PBT would transfer a single *d*-dimensional system to a single (random) Bob; however, the repeated protocol on 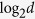 qubits would transfer them across a random distribution of non-locally separated Bobs. The achievable performance of PBT for higher-dimensional quantum system is therefore interesting in its own right.

Here we construct a graphical algebra for computing the explicit performance of PBT based on so-called “pretty-good-measurements”. The graphical representations thus produced have a size which is independent of the Hilbert-space dimension *d*, but is instead related only to the number of ports *N*. This allows us to compute the success probability to distinguish the different outcomes and also the fidelity for PBT even for high dimensions, though currently our analysis is limited to small *N* (we expect that by identifying those graphs with the largest contributions to performance, that we could in future determine the achievable behavior for arbitrary *d* and *N*). The graphical algebra we use is a subalgebra of the Temperley-Lieb algebra and we discuss some of the properties of this subalgebra at the end of the article.

## Results

With our graphical algebra, we have calculated the success probability to distinguish the different outcomes, *S*, and fidelity of PBT for *N* = 2, 3, 4 for arbitrary *d*. We give explicit expressions of the success probability in Eq. (1) and one can obtain the entanglement fidelity, *F*_*e*_, and the average fidelity, *F*, very easily through their relations^1^ *F*_*e*_ = *NS*/*d*^2^ and *F* = (*dF*_*e*_ + 1)/(*d* + 1).


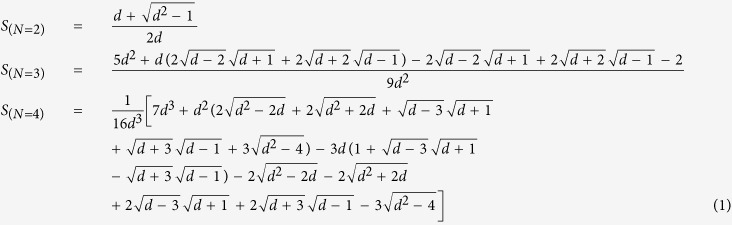


We plot these measures of success probability in Fig. 2. Although only calculated for small *N*, we find that: (i) *S* quickly approaches one as *d* increases; (ii) the entanglement fidelity *F*_*e*_ goes to zero asymptotically with *d* with roughly the exponent of −2; and (iii) the average fidelity *F* is only slightly higher than *F*_*e*_ (note that *F* ≥ *F*_*e*_ must always hold). Across common values of *N* and *d* these results agree with the original analysis of PBT^1^.

We give a trivial upper bond to the entanglement fidelity 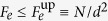 because the success probability is itself upper-bounded by one. A lower bound has been derived^3^ to the entanglement fidelity is 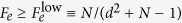. Figure 3 demonstrates that the lower bound corresponds to a good approximation for lower *N* and our trivial upper bound appears to be reached asymptotically for higher dimensions. Note that for a general number of ports satisfying *d* ≫ *N*, we have 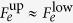; and hence the actual entanglement fidelity for PBT is tightly constrained in these circumstances for asymptotically large dimensionalities.

## Discussion

Our graphical algebra allows us to explicitly determine the higher-dimensional performance of PBT. However, as *N* increases, the size of the algebra will exponentially increase. We tame this growth substantially by relying on the underlying permutation symmetry amongst the ports which allows us to only consider those diagrams within a “conjugacy class”. So far, we have not been able to fully utilize this symmetry in the final steps of the computation of the success probability where the contribution of every member of a conjugacy class needs to be separately evaluated. This difficulty has limited our ability to extend our results beyond *N* = 4, though for arbitrary *d*. It is our expectation that further simplifications can be found to allow our approach to be used for higher *N*; it may even be that the asymptotic behavior can be determined from just the study of a limited number of conjugacy classes. Such an extension will be the subject of future research.

Finally, the properties of the algebra are not yet completely understood. The pretty-good-measurements are determined by a positive operator *ρ* (see methods) whose eigenvalues are of the form *d* ± *i* with *i* < *N*. Naively, for *d* < *N* extra negative eigenvalues would seem to appear in the general expressions constraining *ρ*. In fact, these strictly vanish for any specific *d* < *N* and such terms exactly cancel in the final results for success probability, etc. (see for example Eq. (1)). For these lower dimensionalities this affect seems to be related to singular-perturbation theory, though here with a geometric interpretation where singular behavior corresponds to subspaces which vanish from the problem as *d* is successively decreased. This geometric interpretation of singular-perturbation theory may be new and if so deserves further examination.

## Methods

### Mathematical representation of entanglement fidelity as a graphical algebra

If the teleportation is successful, Bob will discard all of his ports except the *i*th port *B*_*i*_ which ideally will become the unknown quantum state 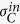. According to the original article on PBT^1^, we may obtain a mathematical expression for the entanglement fidelity for PBT as 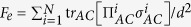, where {Π^*i*^} are the set of POVMs, 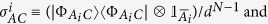 
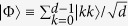
 is the (canonical) maximally entangled state (here 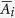 means every system except *A*_*i*_). Details of the derivation are in Supplementary Derivation S1.

According to the above expression for *F*_*e*_, the key part needed to determine the entanglement fidelity of PBT is to calculate the trace of 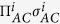. For the choice of so-called “pretty-good measurements”^1^, 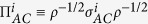 where 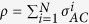. Therefore, the key element of the calculation comes down to analysing the properties of 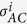. For convenience, we drop the system labelling subscripts and shall use *σ*_*i*_ to denote 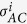, etc., in the following sections.

**Figure 1:**
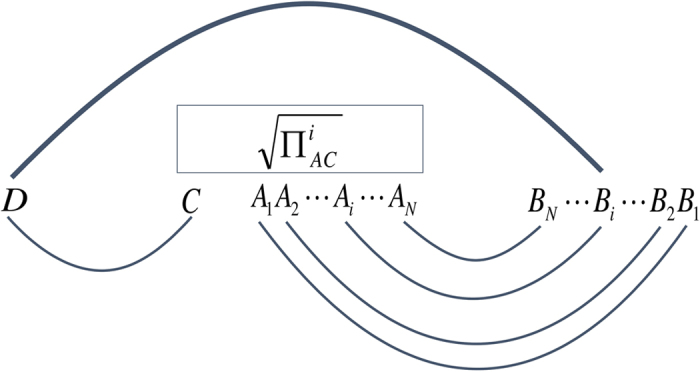
PBT protocol for teleporting entanglement. When the input system *C* is half of an entangled state *σ*_*DC*_, PBT will ‘swap’ the entanglement so that now systems *D* and *B*_*i*_ (corresponding to port *i* in Bob’s possession) become entangled. The joint quantum state *σ*_*DC*_ will have been transferred to 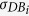. Finally, if *σ*_*DC*_ is initially maximally entangled, then the ‘global’ fidelity (including the entanglement with system *D*) of this teleportation is given by the so-called entanglement fidelity^8^ for this process.

**Figure 2:**
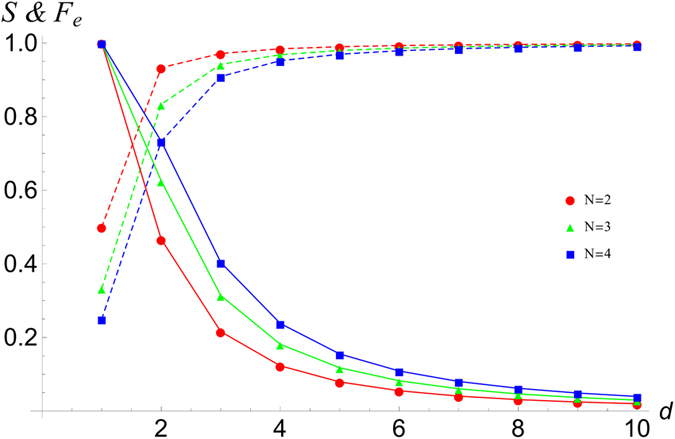
The performance of PBT for N = 2, 3, 4. Here we plot the success probability *S* (dashed lines) and entanglement fidelity *F*_*e*_ (solid lines) of PBT for *N* = 2, 3, 4 as a function of dimension *d*. As *d* increases, the success probability for the POVM approaches one, for increasing *N* we find this success probability decreases.

**Figure 3:**
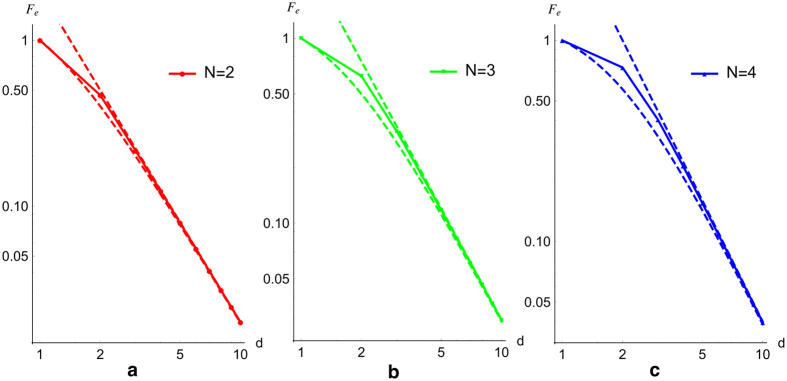
The entanglement fidelity and its upper and lower bounds of PBT for small N. Here we give a log-log plot of *F*_*e*_ versus *d* plot for *N* = 2, 3, 4 computed using our graphical-algebraic techniques. The analytic form for the upper and lower bounds are given in the text. As *d*^2^ ≫ *N* increases, the lower bound asymptotes towards the straight-line upper bound with gradient −2 in this log-log plot.

**Figure 4:**
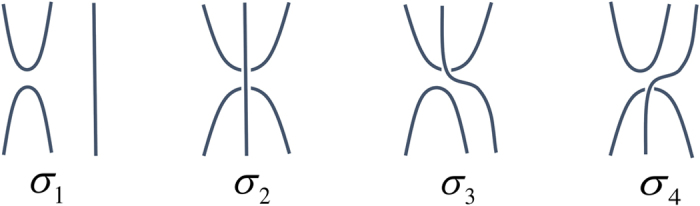
The graph algebra for N = 2. When *N* = 2, we may write down a ‘graphical basis’ for analyzing PBT which we denote for simplicity here as *σ*_1_, *σ*_2_, *σ*_3_, *σ*_4_. Note that to describe *ρ* we need only the first two of these graphs, however, to describe arbitrary powers of *ρ* we will in general require all of these graphs.

Inspired by Fig. 1, we constructed a graphical algebra to represent *σ*_*i*_ in a manner independent of *d*. Let us explain the graphical algebra for the case of two ports, when *N* = 2. The shape ∪ will denote the (unnormalized) ket |Φ〉; the shape ∩ will denote the (unnormalized) ket 〈Φ|, a vertical line will denote the identity operator. So, in Fig. 4, *σ*_1_ denotes the operator |*jji*〉〈*kki*| = |*jj*〉〈*kk*| ⊗ |*i*〉〈*i*| (where there is an implied sum over the repeated indices). Thus, the graph denoted *σ*_1_ corresponds to the (unnormalized) maximally entangled state on subsystems 0 and 1 and the maximally mixed state on subsystem 2. The left-most subsystem in our graphs is labelled zero and permutations of it do not contribute to the algebra. Were we to ‘normalize’ this graph, we could multiply it by 1/*d*^*N*^.

**Figure 5:**
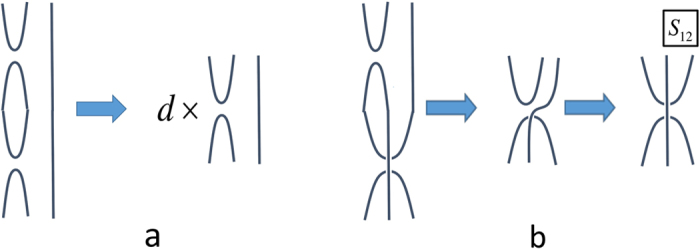
The multiplication rule when N = 2. (**a**) Here we illustrate the multiplication *σ*_1_*σ*_1_. Loops reduce to a numerical factor *d*; (**b**) Here we illustrate the multiplication *σ*_1_*σ*_2_. Lines may be internally rearranged so long as the ordering (or labelling) of all line ends is unchanged. Note that standard forms can be produced by at most the pre- or post-application of permutations among the ports (i.e., excluding subsystem zero). The result of the multiplication is found to be labelled as *σ*_4_ in Fig. 4. This in turn may be written in terms of *σ*_2_ as *S*_12_*σ*_2_ (here *S*_12_ is a simple permutation operator which swaps ports 1 and 2).

Since the graphs represent operators, they may be added and multiplied like operators. Specifically, to multiply two operators denoted by graphs, the graph for the leftmost operator is placed above that of the rightmost operator and the lines of their respective subsystems are joined in the natural manner (see Fig. 5). Any loop reduces to a trace of the identity operator on a *d*-dimensional subsystem, i.e., the factor *d*. Other simplifications come from internally rearranging the lines. Finally, the resultant operators can always be placed into a standard form by at most pre- and post-multiplication by permutation operators on the ports (i.e., those subsystems in Bob’s hand labelled 1, …, *N*). Some of these rules are shown in Fig. 5 for *N* = 2.

The multiplication rules can be summarized by Eq. (2)


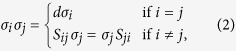


all for *i*, *j* ∈ {1, 2}. All other multiplications may be obtained by noting that *S*_12_*S*_12_ reduces to the the trivial (identity) permutation.

With the above results, we may graphically evaluate the success probability as 
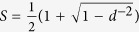
. The details of the derivation are given in Supplementary Derivation S2. For higher *N* we must allow for an accumulation of swap operations on any pair of ports. When combined, these swaps form arbitrary permutations as we shall now consider.

### Graphical Algebra for *N* > 2

Although the result for *N* = 2 is very simple, when we use our approach for larger *N* the size of the algebra increases very quickly. In order to compute the success probability using pretty-good-measurements we can restrict ourselves to fully permutation symmetric expressions. For example the quantity 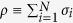 appears in the form *ρ*^−1/2^. By relying on permutation symmetry we may eliminate from active consideration any pre-applied permutation of our graphs. However, this still leaves with an effectively exponential number of graphs differing by a post-applied permutation.

Our main strategy relies on the fact that the algebra generated solely from *ρ* (consisting of the set of elements formed from the series *ρ*, *ρ*^2^, *ρ*^3^, …) must eventually close for any finite *N*. Because of this we will be able to express any power of *ρ* (such as *ρ*^−1/2^) as a finite order polynomial in *ρ* itself. Further, because every element in this algebra commutes with every other element, we may write out the minimal ‘polynomial of closure’ directly in the basis where *ρ* is diagonal. In this way, this polynomial automatically corresponds to a polynomial whose solutions correspond to the eigenvalues of *ρ*. Note that given these eigenvalues we can immediately represent any power of *ρ*, and in particular *ρ*^−1/2^, as a polynomial of this algebra generated from *ρ* itself. See Supplementary Derivation S3 for proofs for these statements.

**Figure 6:**
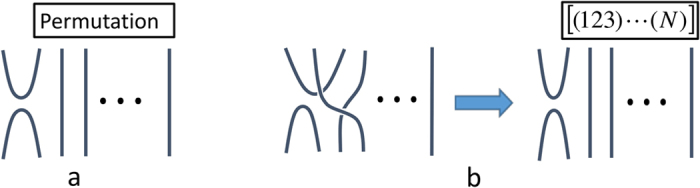
The graphical algebra with permutations for larger N. (**a**) We express permutations by the conventional ‘cycle’ notation of the permutation group. Taking into account the global permutation symmetry every operator in our algebra can be summarized by a sum over post-permutations on *σ*_1_. (**b**) Shows an example expressed as a simple three-cycle [(123) … (*N*)]*σ*_1_.

Computing the closure polynomial involves (sums over) simple repeated multiplications of terms like *σ*_*i*_*σ*_*j*_. Each such multiplication will reduce to a generic term like *S*_*ij*_*σ*_*j*_. Iterating this leads to a sequence of post-swap operations, or equivalently a single post-permutation operation. This means that a typical term from *ρ*^*n*^ will consist of a sum over terms shown in Fig. 6. We denote permutations using the conventional cyclic notation, so that (123) ... (*N*) denotes shifting 1 → 2, 2 → 3 and 3 → 1, etc. To represent this as a unitary operator permuting the ports we surround it by square brackets, e.g., [(123) ... (*N*)].

To further reduce the complexity of our results we can further rely on global permutation symmetry to ensure that once one term with a specific ‘class’ of permutations appears, e.g., a single three cycle such as for the permutation (123)(4) ... (*N*), then every three-cycle permutation up to port relabelling will also appear (equivalence under relabelling yields an equivalence class of permutations). By separately being able to compute the size of such conjugacy classes we need only explicitly store the appearance of an individual exemplar from each class when it appears. The computation of the size of these conjugacy classes is given explicitly in the next section.

The polynomial expressions for *ρ*^−1/2^ involve coefficients which explicitly depend on *d*. Finally, we can use our graphical representation to simplify and explicitly compute the success probability which is given by tr(*ρ*^−1/2^*σ*_*i*_*ρ*^−1/2^*σ*_*i*_). Computing the trace is easy. The terms are multiplied, and put into a sum over the standard form [Permutation]*σ*_1_; for each graph we close and join the *i*th line at the top with the same line at the bottom and then count loops — each loop yielding a factor of *d*. For example tr{[(12)(3)(4)]*σ*_*i*_} = *d*^3^. This allows us to directly compute the success probability and as already mentioned the entanglement fidelity and average fidelity then immediately follow. We explain our calculation with more details in Supplementary Derivation S4.

All calculations we performed using Mathematica.

### Conjugacy Classes

The number of conjugacy classes for different *N* is very useful because it provides an upper bound on the number of distinct eigenvalues of the operator *ρ* and can tell us how large the algebra can be. We find the number of conjugacy classes can be determined by a variation of integer partition theory.

Firstly, let us review conventional integer partition theory. A partition of a positive integer *n* is a way of writing *n* as a sum of positive integers without regard to the order in which the sum is written out^9^.The number of partitions of an integer *n* is given by the so-called partition function *P*(*n*). The partition function *P*(*n*) may be most easily expressed in terms of its generating function as


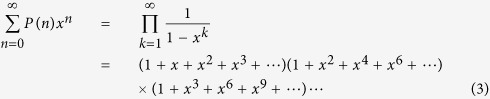


The partition function is interesting for us because it tells us the number of conjugacy classes of permutations that exist (i.e., have the same form up to global relabelling).

Since, in our graphs, one of the ports corresponds to a special object (the port ultimately entangled with Alice’s system zero) we must distinguish our conjugacy classes depending on where within the permutation this special object sits. To count the number of permutations with a distinct form (taking into account this special object) we must consider a variation of the conventional integer partition theory. For example, when *n* = 3, the partition 2 + 1 would become two partitions when one takes into account that our special object may sit either in the “1” or the “2”. By contrast, the partition 1 + 1 + 1 remains a single partition, since although only one of the “1”s contain our special object the ordering in the sum makes no difference. As already mentioned every partition corresponds to a distinct permutation conjugacy class. The number of these classes is then given by the integer partition function, *W*(*n*), for our variation of this problem. Note, that the existence of a special object can only increase the number of partitions, so we have the trivial lower bound *P*(*n*) ≤ *W*(*n*).

If we think about temporarily labelling our special object as *y* then it is not hard to see that 
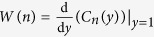
, where


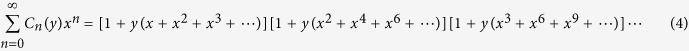


Expanding this allows us to express our generating function directly as a function of *x* by


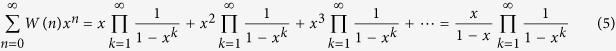


We finally note that by dividing both sides of the Eq. (5) by *x*, and replacing the right-hand-side of the result by Eq. (3), we may obtain


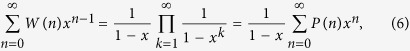


which proves that 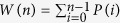. From the generating function of *W*(*n*) we may also obtain an upper bound for our partition function as *W*(*n*) ≤ 1 + *nP*(*n* − 1)/2. We prove this in Supplementary Derivation S5.

## Additional Information

**How to cite this article**: Wang, Z.-W. and Braunstein, S. L. Higher-dimensional performance of port-based teleportation. *Sci. Rep.* **6**, 33004; doi: 10.1038/srep33004 (2016).

## Supplementary Material

Supplementary Information
