# Supplementary material for: Higher-dimensional performance of port-based teleportation
Source: Sci Rep. 2016 Sep 8;6:33004. doi: 10.1038/srep33004 (PMC5015100; doi:10.1038/srep33004)
Supplement: Supplementary Information [file srep33004-s1.pdf]

Supplementary information
Higher-dimensional performance of port-based teleportation

Zhi-Wei Wang[1,*] and Samuel L. Braunstein[2,+]

[1]Tang Aoqing Honors Program in Science, College of Physics, Jilin University, Changchun, 130012, People's Republic of China
[2]Computer Science and York Centre for Quantum Technologies, University of York, York YO10 5GH, United Kingdom
[*]phyjasonwang@gmail.com
[+]sam.braunstein@york.ac.uk

Inventory of Supplemental Information

1. **Supplementary Derivation S1**
   - Mathematical representation of the entanglement fidelity

2. **Supplementary Derivation S2**
   - Performance of PBT when $N = 2$

3. **Supplementary Derivation S3**
   - Minimal polynomial of ρ

4. **Supplementary Derivation S4**
   - The calculations method for larger N

5. **Supplementary Derivation S5**
   - Upper bound of the number of the conjugacy class

Notation: In the following paragraphs, we use the same symbols as in the formal paper.

Supplementary Derivation S1

If the teleportation is successful, Bob will discard all of his ports except the ith port B_i which is the unknown quantum state σ_C^{in}. We derive the mathematical representation of the entanglement fidelity for PBT according to Satoshi Ishizaka and Tohya Hiroshima's initial article[1].

$$\begin{aligned}
\sigma^{\text{out}} &= \Lambda(\sigma_C^{\text{in}}) \\
&= \sum_{i=1}^{N} tr_{A\bar{B}_iC}\left[\sqrt{\Pi_{AC}^i}\left(\bigotimes_{i=1}^{N}|\Phi_{A_iB_i}\rangle\langle\Phi_{A_iB_i}|\otimes\sigma_C^{\text{in}}\right)\sqrt{\Pi_{AC}^i}\right] \\
&= \sum_{i=1}^{N} tr_{AC}\left[\sqrt{\Pi_{AC}^i}\left(\sigma_{AB_i}^i\otimes\sigma_C^{\text{in}}\right)\sqrt{\Pi_{AC}^i}\right]
\end{aligned} \tag{1}$$

In the last step, we use the notation: $\sigma_{AB_i}^i = tr_{\bar{B}_i}(\otimes_{i=1}^N|\Phi_{A_iB_i}\rangle\langle\Phi_{A_iB_i}|) = 1/d^{N-1}|\Phi_{A_iB_i}\rangle\langle\Phi_{A_iB_i}|\otimes\mathbb{1}_{\bar{A}_i}$. $\bar{B}_i$ means every port state except B_i, $\bar{A}_i$ means every state except A_i, and $\langle\Phi| = 1/\sqrt{d}\sum_{k=0}^{d-1}\langle kk|$ is the maximally entangled state.

For the entanglement fidelity, the input state σ_C^{in} should be half part of a maximally entangled state $|\Phi_{DC}\rangle\langle\Phi_{DC}|$, like Fig.1 in the article. Then Eq. (1) can be translated into $\sigma^{\text{out}} = \sum_{i=1}^N tr_{AC}\left[\Pi_{AC}^i(\sigma_{AB_i}^i\otimes|\Phi_{DC}\rangle\langle\Phi_{DC}|)\right]$, and the entanglement fidelity should be:

$$\begin{aligned}
F_e &= \sum_{i=1}^{N}\langle\Phi_{DB_i}|tr_{AC}\left[\Pi_{AC}^i\left(\sigma_{AB_i}^i\otimes|\Phi_{DC}\rangle\langle\Phi_{DC}|\right)\right]|\Phi_{DB_i}\rangle \\
&= \sum_{i=1}^{N} tr_{AC}\left[\Pi_{AC}^i\left(\langle\Phi_{DB_i}|\Phi_{DC}\rangle\otimes\sigma_{AB_i}^i\otimes\langle\Phi_{DC}|\Phi_{DB_i}\rangle\right)\right]
\end{aligned} \tag{2}$$

Term $\langle\Phi_{DC}|\Phi_{DB_i}\rangle$ can be translated into:

$$\begin{aligned}
&\langle\Phi_{DC}|\Phi_{DB_i}\rangle \\
&= \frac{1}{d}\sum_{l,m=0}^{d-1}(\langle l_D|\langle l_C|)(|m_D\rangle|m_{B_i}\rangle) \\
&= \frac{1}{d}\sum_{l=0}^{d-1}|l_{B_i}\rangle\langle l_C|
\end{aligned} \tag{3}$$

With Eq. (3), Eq. (2) can be translated into:

$$\begin{aligned}
F_e &= \sum_{i=1}^{N}\frac{1}{d^2}tr_{AC}\left[\Pi_{AC}^i\left(\sum_{l,k=0}^{d-1}|l_C\rangle\langle l_{B_i}|\otimes\sigma_{AB_i}^i\otimes|k_{B_i}\rangle\langle k_C|\right)\right] \\
&= \sum_{i=1}^{N}\frac{1}{d^2}tr_{AC}\left[\Pi_{AC}^i\sigma_{AC}^i\right]
\end{aligned} \tag{4}$$

In the last step, we use $\sum_{l=0}^{d-1}|l_C\rangle\langle l_{B_i}|\Phi_{A_iB_i}\rangle = |\Phi_{A_iC}\rangle$.

Supplementary Derivation S2

For convenience, we will denote σ_{AC}^i as σ_i in the following paragraphs. For N=2, we have obtained the following equation rules in the paper (denote $Swap_{ij}$ as S_{ij}).

$$\sigma_i\sigma_j = \begin{cases} d\,\sigma_i & \text{if } i = j \\ S_{ij}\,\sigma_j = \sigma_j\,S_{ji} & \text{if } i \neq j \end{cases} \tag{5}$$

When $N=2$, $\rho=\sigma_1+\sigma_2$. We express $\sqrt{\rho}$ as the combination of the four states like Eq. (6), and use relation $\rho=(\sqrt{\rho})^2$ to solve α and γ. As σ_1 and σ_2 share the same position in the algebra, we give them the same coefficient and so do $S_{12}\sigma_1$ and $S_{12}\sigma_2$.

$$\sqrt{\rho}=\alpha[(\sigma_1+\sigma_2)+\gamma(S_{12}\sigma_1+S_{12}\sigma_2)] \tag{6}$$

The solutions for γ and α are:

$$\begin{cases}\gamma_{\pm}=-d\pm\sqrt{(d^2-1)}\\ \alpha^2=\frac{1}{2\gamma(1-d^2)}\end{cases} \tag{7}$$

Then we express $\rho^{-1/2}$ as $\beta[(\sigma_1+\sigma_2)+\tau(S_{12}\sigma_1+S_{12}\sigma_2)]$, and using the equation $\sqrt{\rho}=\rho^{-1/2}\rho$ to solve $\rho^{-1/2}$. The solutions for β and τ are:

$$\begin{cases}\beta=\alpha(\frac{d-\gamma}{d^2-1})\\ \tau=\frac{d\gamma-1}{d-\gamma}\end{cases} \tag{8}$$

So the success probability(normalized) is Eq. (9), the entanglement fidelity[1] is $F_e=(d+\sqrt{d^2-1})/d^3$ and the average fidelity is $F=(F_ed+1)/(d+1)=(d+\sqrt{(d-1)/(d+1)})/d^2$. This result agree with Satoshi Ishizaka and Tohya Hiroshima's initial analyse[1].

$$\begin{aligned}S&=\frac{1}{d^2}tr_{AC}\left[\prod_{AC}^{i}\sigma_i\right]\\&=\frac{1}{d^2}tr(\rho^{-1/2}\sigma_1\rho^{-1/2}\sigma_1)\\&=\frac{1}{d^4}tr(\sigma_1\rho^{-1/2}\sigma_1\sigma_1\rho^{-1/2}\rho_1)\\&=\frac{d+\sqrt{d^2-1}}{2d}\end{aligned} \tag{9}$$

Supplementary Derivation S3

When we consider the linear relation between diagonal matrices, it equals to consider the column vector (C_ρ) formed by the diagonal elements of the matrices. If we assume all the none-zero different diagonal elements of matrices D_ρ (the corresponding diagonal matrices of ρ) is $\{r_1,r_2,r_3,\ldots,r_n\}$, then we can prove $\{C_\rho,(C_\rho)^2,(C_\rho)^3,\ldots,(C_\rho)^n\}$ is independent of each other and $(C_\rho)^{n+1}$ is a linear combination of this set. We denote the matrix $\{C_\rho,(C_\rho)^2,(C_\rho)^3,\ldots,(C_\rho)^n\}$ by δ, and expand its determinant in Eq. (10). The determinant in the second step of Eq. (10) is the Vandermonde determinant[2] of order n which equals to $\prod_{1\le j<i\le n}(r_i-r_j)$. As all the elements r_i are none-zero and different, the final result of Eq. (10) must be none-zero. Thus, matrix δ is full rank and $\{C_\rho,(C_\rho)^2,(C_\rho)^3,\ldots,(C_\rho)^n\}$ must be independent of each other.

$$|\delta|=\begin{vmatrix}r_1 & r_1^2 & r_1^3 & \ldots & r_1^n\\ r_2 & r_2^2 & r_2^3 & \ldots & r_2^n\\ \cdots&\cdots&\cdots&\cdots&\cdots\\ r_n & r_n^2 & r_n^3 & \ldots & r_n^n\end{vmatrix}=\left(\prod_{1\le k\le n}r_k\right)\begin{vmatrix}1 & r_1 & r_1^2 & \ldots & r_1^{n-1}\\ 1 & r_2 & r_2^2 & \ldots & r_2^{n-1}\\ \cdots&\cdots&\cdots&\cdots&\cdots\\ 1 & r_n & r_n^2 & \ldots & r_n^{n-1}\end{vmatrix}=\left(\prod_{1\le k\le n}r_k\right)\left(\prod_{1\le j<i\le n}(r_i-r_j)\right) \tag{10}$$

Next, we will prove $(C_\rho)^{n+1}$ is a linear combination of the lower power of (C_ρ). As matrix δ is full rank, Eq.(11) must have one none-zero solution which means $(C_\rho)^{n+1}$ is the linear combination of set $\{(C_\rho)^i\}$ $(1\le i\le n)$. As column vector C_ρ equals to the diagonal form of matrix ρ, we can replace it by ρ and obtain expression $\rho^{n+1}=a_1\rho+a_2\rho^2+\ldots+a_n\rho^n$ which equals to the minimal polynomial $\rho^n=a_1+a_2\rho+\ldots+a_n\rho^{n-1}$.

$$\begin{pmatrix}r_1 & r_1^2 & r_1^3 & \ldots & r_1^n\\ r_2 & r_2^2 & r_2^3 & \ldots & r_2^n\\ \cdots&\cdots&\cdots&\cdots&\cdots\\ r_n & r_n^2 & r_n^3 & \ldots & r_n^n\end{pmatrix}\begin{pmatrix}a_1\\a_2\\ \vdots\\ a_n\end{pmatrix}=\begin{pmatrix}r_1^{n+1}\\ r_2^{n+1}\\ \vdots\\ r_n^{n+1}\end{pmatrix} \tag{11}$$

Supplementary Derivation S4

We will give more details about the calculations method we use for larger N. Although we use $N=2$ as an example to explain the method, the method is general.

We firstly calculate $\rho,\rho^2,\rho^3,\dots$ based on the permutation theory. We find all kinds of conjugacy classes already appear in ρ^2, which means the algebra is close at ρ^2. Thus, we can solve $\rho^3=a_1\rho+a_2\rho^2$ based on their permutation expressions and the solution is $a_1=1-d^2, a_2=2d$. Therefore, the minimal polynomial of ρ is $\rho^2=1-d^2+2d\rho$ whose solutions are ρ's different nonzero eigenvalues $d-1$ and $d+1$. Using these eigenvalues, we can built up the support matrix of ρ,ρ^2 and directly calculate the support matrix of $\rho^{-1/2}$. According to the support matrix, $\rho^{-1/2}=b_1\rho+b_2\rho^2$ while b_1 and b_2 are expressions about d. As long as we insert the permutation expression of ρ back into this relation we can get the permutation expression of $\rho^{-1/2}$. Finally, we can calculate $\rho^{-1/2}\sigma_1\rho^{-1/2}\sigma_1$ and its trace based on the permutation expression. The result is $(d+\sqrt{d^2-1})/2d$ which agrees with the result in the Supplementary Derivation S2.

Because all the calculation is based on the permutation expression, so the essential part is to figure out how the permutation states interact with each other. As there is an entanglement element in the permutation, the two permutation cannot directly multiply with each other like traditional permutation. We will denote $Permutation_i$ as P_i and explain its special multiply rules.

Combining with the discussion in the paper, we know $(P_i\sigma_i)*(P_j\sigma_j)$ is the general form of the multiplication which equals to $P_k\sigma_j$. To figure out P_k, we only need change P_j into a new permutation and directly multiply P_i with the new P_j like traditional permutation. To figure out the new P_j, we obtain the following three rules through analysing the graphical algebra. Notice, every parenthesis represents a circle of all the numbers in it; and A,B,C,D represent a series of number.

(1) If i and j are in different circles, then we only need to find which number will arrive i and connect it to the number after j, like Eq. (12).

$$\begin{aligned}&P_i\sigma_i*[(AiB)(CjD)(\cdots)\cdots]\sigma_j\\=&P_i*[(iBADCj)(\cdots)\cdots]\sigma_j\\=&P_k\sigma_j\end{aligned}\tag{12}$$

(2) If i and j are in the same circle, then cut out the part from i to j and make it a separate circle and remind all the other unchanged, like Eq. (13).

$$\begin{aligned}&P_i\sigma_i*[(AiBjC)(\cdots)\cdots]\sigma_j\\=&P_i*[(iBj)(AC)(\cdots)\cdots]\sigma_j\\=&P_k\sigma_j\end{aligned}\tag{13}$$

(3) If the number before i is j, then add d, like Eq. (14). In fact, this is the condition that the graphical algebra will produce a circle.

$$\begin{aligned}&P_i\sigma_i*[(AjiB)(\cdots)\cdots]\sigma_j\\=&dP_i*[(AjiB)(\cdots)\cdots]\sigma_j\\=&dP_k\sigma_j\end{aligned}\tag{14}$$

We write all the codes in Mathematica based on the above method.

During calculating the minimal polynomial, we use the conjugacy class to simplify the expression of $\rho,\rho^2,\dots$, and during calculating the trace of $\rho^{-1/2}\sigma_1\rho^{-1/2}\sigma_1$ we have to return to the full terms format. The number of terms making up the state for $N=2,3,4,5$ separately is $4,18,96,600$ while the corresponding conjugacy class number is $2,4,7,12$. So conjugacy class theory can exponentially simplify our calculation in some steps.

Supplementary Derivation S5

We want to make a little change on the generating function of the traditional partition like Eq. (15) and obtain $P(n) = 1 + a_2 + \cdots + a_n$ ($a_i \leq a_j$ when $i < j$).

$$\begin{aligned}
&\sum_{n=0}^{\infty} P(n)x^n \\
=&(1+x+x^2+x^3+\cdots)(1+x^2+x^4+x^6+\cdots)(1+x^3+x^6+x^9+\cdots)\cdots \\
=&(1+x+x^2+x^3+\cdots)(1+a_2x^2+a_3x^3+a_4x^4+\cdots) \\
=&\sum_{n=0}^{\infty}(1+a_2+\cdots+a_n)x^n
\end{aligned} \tag{15}$$

The result of Eq. (15) can translate the final generating function $W(n) = \sum_{i=0}^{n-1} P(i)$ into Eq. (16). As $a_i \leq a_j$ when $i < j$, we must have $W(n) \leq \frac{n}{2}P(n-1) + 1$.

$$\begin{aligned}
W(n) =&1 \\
&+1 \\
&+1+a_2 \\
&+1+a_2+a_3 \\
&+1+a_2+a_3+a_4 \\
&\quad\cdots \\
&+1+a_2+a_3+\cdots+a_{n-1} \\
&\leq \frac{n}{2}P(n-1)+1
\end{aligned} \tag{16}$$

References

1. Ishizaka, S. & Hiroshima, T. Asymptotic teleportation scheme as a universal programmable quantum processor. *Physical review letters* **101**, 240501 (2008).
2. Sobczyk, G. Generalized vandermonde determinants and applications. *Aportaciones Matematicas, Serie Comunicaciones* **30**, 203–213 (2002).
